# UK Lung Cancer RCT Pilot Screening Trial: baseline findings from the screening arm provide evidence for the potential implementation of lung cancer screening

**DOI:** 10.1136/thoraxjnl-2015-207140

**Published:** 2015-12-08

**Authors:** J K Field, S W Duffy, D R Baldwin, D K Whynes, A Devaraj, K E Brain, T Eisen, J Gosney, B A Green, J A Holemans, T Kavanagh, K M Kerr, M Ledson, K J Lifford, F E McRonald, A Nair, R D Page, M K B Parmar, D M Rassl, R C Rintoul, N J Screaton, N J Wald, D Weller, P R Williamson, G Yadegarfar, D M Hansell

**Affiliations:** 1 Roy Castle Lung Cancer Research Programme, Department of Molecular and Clinical Cancer Medicine, University of Liverpool, Liverpool, UK; 2 Queen Mary University of London, London, UK; 3 Department of Respiratory Medicine, Nottingham University Hospitals, Nottingham, UK; 4 School of Economics, University of Nottingham, Nottingham, UK; 5 Royal Brompton and Harefield NHS Foundation Trust, London, UK; 6 Cardiff University School of Medicine, Cardiff, UK; 7 University of Cambridge, Cambridge Biomedical Research Centre, Cambridge, UK; 8 Department of Pathology, Royal Liverpool and Broadgreen University Hospital Trust, Liverpool, UK; 9 Liverpool Heart and Chest Hospital, NHS Foundation Trust, Liverpool UK; 10 Lung Cancer Patient Advocate, Liverpool, UK; 11 Department of Pathology, Aberdeen Royal Infirmary, Aberdeen, UK; 12 Guy's and St Thomas’ NHS Foundation Trust, London, UK; 13 Medical Research Council Clinical Trials Unit at UCL, London, UK; 14 Department of Histopathology, Papworth Hospital NHS Foundation Trust, Cambridge, UK; 15 Center for Population Health Sciences, University of Edinburgh, Edinburgh, UK

**Keywords:** Lung Cancer, Imaging/CT MRI etc

## Abstract

**Background:**

Lung cancer screening using low-dose CT (LDCT) was shown to reduce lung cancer mortality by 20% in the National Lung Screening Trial.

**Methods:**

The pilot UK Lung Cancer Screening (UKLS) is a randomised controlled trial of LDCT screening for lung cancer versus usual care. A population-based questionnaire was used to identify high-risk individuals. CT screen-detected nodules were managed by a pre-specified protocol. Cost effectiveness was modelled with reference to the National Lung Cancer Screening Trial mortality reduction.

**Results:**

247 354 individuals aged 50–75 years were approached; 30.7% expressed an interest, 8729 (11.5%) were eligible and 4055 were randomised, 2028 into the CT arm (1994 underwent a CT). Forty-two participants (2.1%) had confirmed lung cancer, 34 (1.7%) at baseline and 8 (0.4%) at the 12-month scan. 28/42 (66.7%) had stage I disease, 36/42 (85.7%) had stage I or II disease. 35/42 (83.3%) had surgical resection. 536 subjects had nodules greater than 50 mm^3^ or 5 mm diameter and 41/536 were found to have lung cancer. One further cancer was detected by follow-up of nodules between 15 and 50 mm^3^ at 12 months. The baseline estimate for the incremental cost-effectiveness ratio of once-only CT screening, under the UKLS protocol, was £8466 per quality adjusted life year gained (CI £5542 to £12 569).

**Conclusions:**

The UKLS pilot trial demonstrated that it is possible to detect lung cancer at an early stage and deliver potentially curative treatment in over 80% of cases. Health economic analysis suggests that the intervention would be cost effective—this needs to be confirmed using data on observed lung cancer mortality reduction.

**Trial registration:**

ISRCTN 78513845.

**Key messages**

**What is the key question?**

- Is lung cancer CT screening a viable option in the UK and is it cost effective?

**What is the bottom line?**

- A single low-radiation dose CT screen in people at high risk of lung cancer as selected by a validated risk prediction model led to a 2.1% detection rate with 86% stage I and II and an 83% resection rate.

**Why read on?**

- The UK Lung Cancer Screening pilot trial is the first to use a true population approach to selection with a validated individual risk assessment and shows that, with volumetric nodule measurement and pre-specified clinical pathways, it is possible to detect lung cancer at an early stage and deliver potentially curative treatment in over 80% of cases: health economic analysis suggests that the intervention would be cost effective, but this needs to be confirmed using data on observed lung cancer mortality reduction.

## Introduction

Almost three-quarters of people with lung cancer present with advanced stage disease, when treatment has little effect on survival. In 2010 the US-based National Lung Cancer Screening Trial (NLST) was stopped 1 year early because a 20% relative reduction in lung cancer mortality had been achieved by low-dose CT compared with chest x-ray after three annual screens and 6 years of follow-up.^1^ Subsequent publications have identified a number of areas where improvements can be made.^2^ Six recommendations were made in the International Association for the Study of Lung Cancer (IASLC) CT screening workshop report,^3^ with a focus on future implementation. These were:

1. Optimisation of identification of high-risk individuals.
2. Development of radiological guidelines.
3. Development of guidelines for the clinical workup of indeterminate nodules.
4. Development of guidelines for pathology reporting.
5. Definition of criteria for surgical and therapeutic interventions of suspicious nodules identified through lung cancer CT screening programmes.
6. Development of recommendations for the integration of smoking cessation practices into future national lung cancer CT screening programmes.

Resolution of these issues is important to ensure that future national lung cancer screening programmes target a population at high enough risk of developing lung cancer while minimising the potential for harm, in a cost-effective way.^4^

The aims of the UK Lung Cancer Screening (UKLS) pilot trial were to demonstrate the effectiveness of risk prediction modelling for the selection of high-risk participants; to evaluate the use of volumetric analysis in the management of CT-detected nodules linked with pragmatic follow-up strategies; and to determine cost effectiveness based on modelling of the pilot UKLS approach.

## Methods

The UKLS trial received approval from the National Information Governance Board. Ethical approval for the study was given by Liverpool Central Research Ethics Committee in December 2010 (reference number 10/H1005/74). The trial was registered with the International Standard Randomised Controlled Trial Register under the reference 78513845.

The UKLS is a randomised controlled trial of low-dose CT (LDCT) screening versus standard care for the early detection of lung cancer in high-risk individuals. The study follows the Wald Single Screen Design, as described previously.^5^

### Sample size

We designed the pilot to give a precise estimate of the proportion of subjects complying with screening. A study size of 4000 and therefore 2000 in the intervention arm would enable us to estimate 80% compliance with a 95% CI not exceeding ±2%.

### UKLS trial design

The Wald single-screen design was adopted because it is the most economical approach in terms of the number of CT screening examinations needed for a fully powered trial^5^ (as it was originally planned that 32 000 individuals were to be randomised in the main UKLS trial). It will also provide early data on rates of cancers in the years following a screen, to inform the most appropriate ‘interval’ for subsequent screens. It will produce mortality results in a similar timeframe to the other major international multicentre screening trials, and allow us to synchronise our data. In addition, the single screen design does not have the problem of long-term compliance.

### Identification of Individuals with a high risk of developing lung cancer

The eligibility criteria in the current two largest randomised controlled trials (NLST and NELSON) were based on smoking history and age,^1^ ^6^ However, risk of lung cancer is also influenced by other factors that can be used to refine risk prediction and improve selection.

The Liverpool Lung Project (LLP) risk model was based on a case–control study.^7^ It is a multivariable conditional logistic regression model based on factors significantly associated with lung cancer (smoking duration, prior diagnosis of pneumonia, occupational exposure to asbestos, prior diagnosis of malignant tumour and early onset (<60 years) family history of lung cancer).^7^ The multivariable model was combined with age-standardised incidence data to estimate the absolute risk of developing lung cancer. The discrimination of the LLP was evaluated and demonstrated its predicted benefit for stratifying patients for CT screening by using data from three independent studies from Europe and North America.^8^

The LLP_v2_ model includes all respiratory disease (COPD, emphysema, bronchitis, pneumonia and TB^9^) and all smokers (cigarettes, pipe and cigars) to select subjects with ≥5% risk of developing lung cancer in the following 5 years.^10^ The risk prediction model is available on http://www.MylungRisk.org.

### Inclusion and exclusion criteria

#### Inclusion criteria

A 5-year lung cancer risk of ≥5%, based on the LLP_v2_ risk prediction model,^7^ ^8^ ^10^ men and women aged between 50 and 75 years old, ability to provide fully informed written consent.

#### Exclusion criteria

Inability to give consent, comorbidity which would unequivocally contraindicate either screening or treatment if lung cancer were detected, thoracic CT performed within 1 year preceding the invitation to be screened, inability to lie flat.

### Recruitment

**Figure 1:**
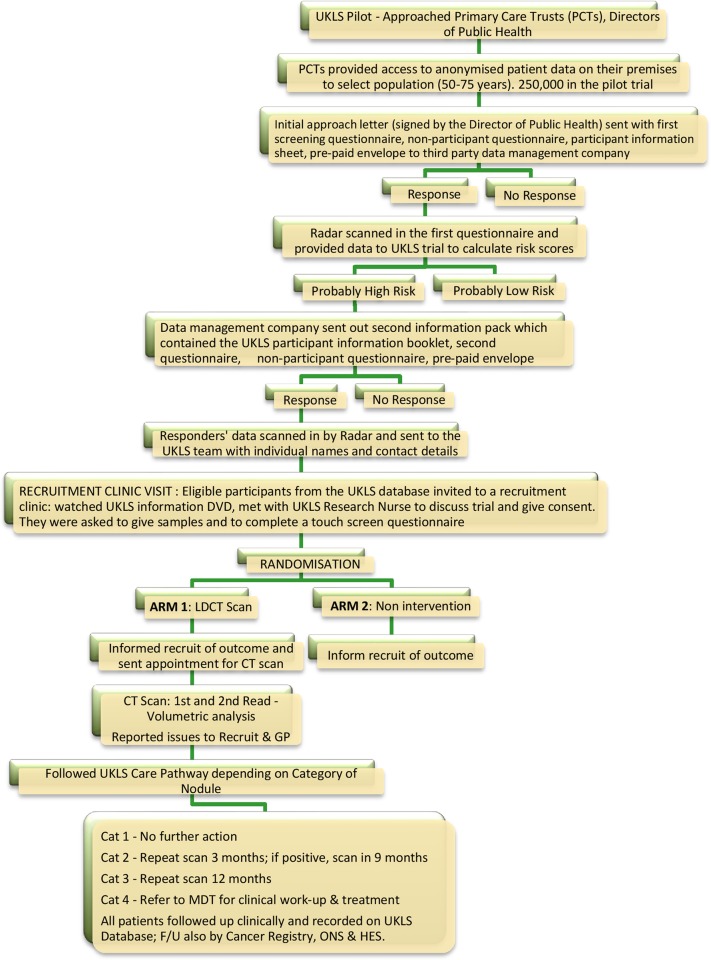
UK Lung Cancer Screening (UKLS) trial recruitment and implementation process. LDCT, low-dose CT scan; MDT, multidisciplinary team.

We identified 249 988 individuals from population Primary Care Trust records, aged 50–75 years, residing in specific healthcare areas (Liverpool, Knowsley, Sefton; Cambridgeshire, Peterborough, and Bedfordshire). Questionnaires were sent to 247 354 individuals to identify those at high risk (≥5% over 5 years) of developing lung cancer. The first UKLS approach questionnaire is available on the UKLS website (http://www.UKLS.org) and was distributed by Radar, the third party data management company (http://www.marketingradar.com). A second questionnaire was sent to individuals considered to be at high risk, inviting them to participate in the UKLS trial (http://www.UKLS.org). Recruited subjects were randomly allocated by simple computer pseudo-random number generation to either the intervention (LDCT) or control arms in a 1:1 ratio. Figure 1 illustrates the trial recruitment process.

### CT

CT scans were performed on Siemens 128-slice scanners (Siemens, Erlangen, Germany) at Papworth Hospital, Cambridgeshire and Liverpool Heart and Chest Hospital (LHCH). In the initial phase of the trial, from November 2011 to December 2011, a Philips Brilliance 64 slice scanner (Philips, Best, The Netherlands) was used at LHCH, using the same acquisition parameters.

Thoracic CT images were obtained from lung apices to bases, during suspended inspiration, in a single breath hold and without the administration of intravenous contrast. Images were reconstructed at 1 mm thickness at 0.7 mm increments, using a moderate spatial frequency kernel reconstruction algorithm. Acquisition parameters (kVp and mAs) varied according to body habitus to achieve a CT dose index below 4 milliGray.

### Reading methods

To optimise nodule detection, all baseline CTs were read by two experienced thoracic radiologists, one at the local trial centres (LHCH or Papworth Hospital) and one at a central site (Royal Brompton Hospital (RBH)). All discrepancies were reviewed by a third thoracic radiologist at RBH, who was the final arbiter. Once consensus had been reached, a letter was sent to the participant and their GP giving the results of the CT.

**Figure 2:**
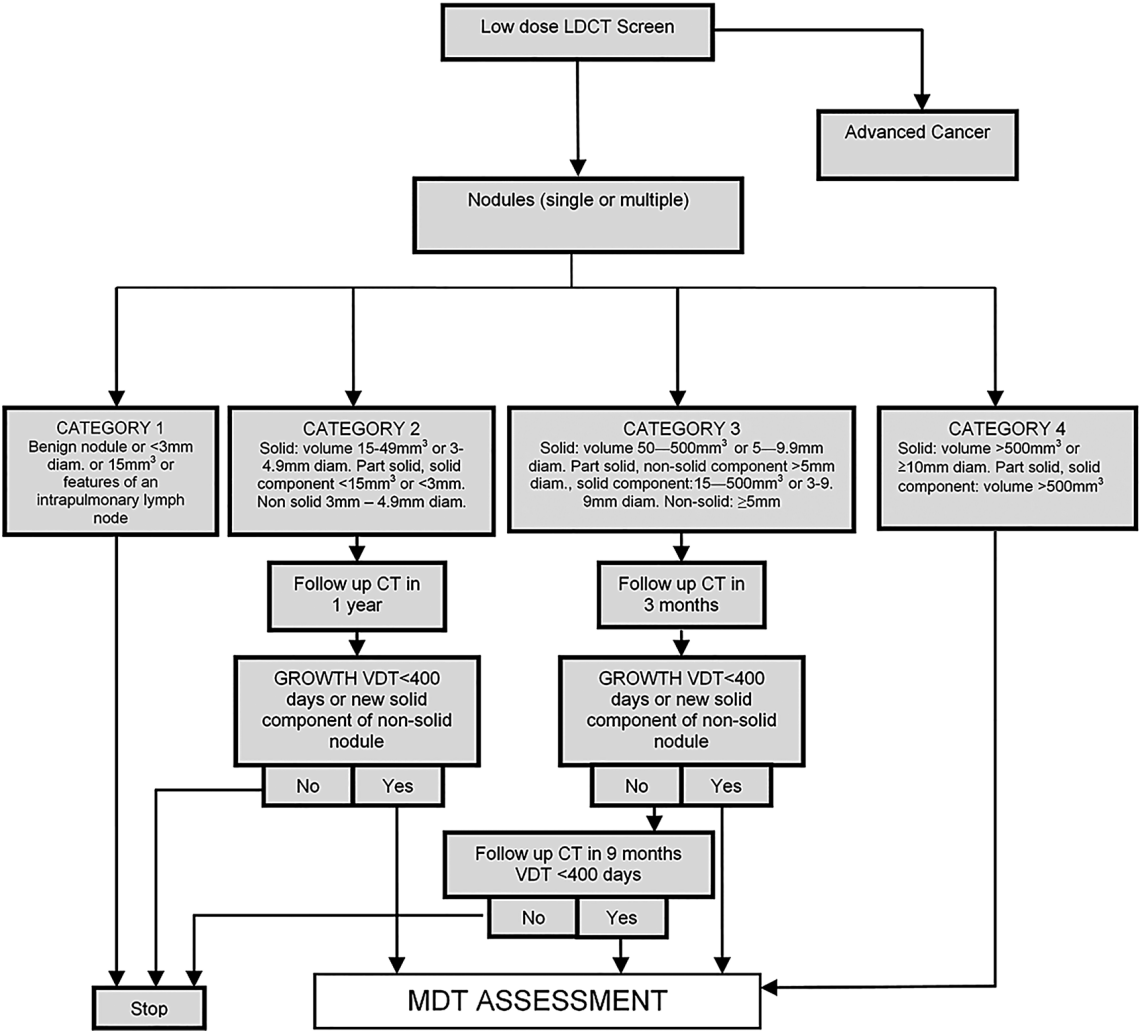
UK Lung Cancer Screening (UKLS) nodule care pathway management protocol. LDCT, low-dose CT scan; MDT, multidisciplinary team; VDT, volume doubling time.

Readers were required to identify and record all lung nodules greater than 15 mm^3^ or 3 mm in maximum diameter (when volumetry was not possible) as per the UKLS nodule management protocol (figure 2). CTs were read using nodule volumetry software (Siemens Syngo LungCare). Maximum intensity projections (MIPs) were used to aid detection.

### Nodule management

Classification of CT findings was based on the UKLS radiology protocol, utilising nodule diameter and volume, on the Siemens LungCARE software platform. Nodule management is shown in figure 2; a summary is given below:

- No nodules or category 1 (benign) nodules: no further action required.
- Category 2 (small, probably benign) nodules: follow-up CT scan at 12 months.
- Category 3 (larger, potentially malignant) nodules: follow-up CT scan at 3 months and 12 months.
- Category 4 (higher chance of malignancy) nodules: immediate referral to multidisciplinary team (MDT).

Nodules greater than 500 mm^3^ or 10 mm maximum diameter at baseline (category 4) or nodules that demonstrated growth on follow-up CT (as defined by a volume doubling time <400 days) were referred to the local MDT for further assessment.

The nodule management protocol has many similarities to that used by the NELSON trial, except that a cutoff volume of 15 mm^3^ was specified to minimise the risk of missing small lung cancers within the single screen design.

### Assessment of harms

All procedures performed by the local MDTs were recorded along with any complications. Detailed psychological assessments were made at intervals during the study; these are the subject of separate planned publications.

### The proposed outcomes of the pilot UKLS Trial

- Population-based recruitment based on risk stratification.
- Trial management through a web-based database.
- Define optimal characteristics of CT readers (radiologists vs radiographers).
- Characterisation of CT-detected nodules utilising volumetric analysis.
- Prevalence of lung cancer at baseline.
- Socio-demographic factors affecting participation.
- Psychosocial measures (cancer distress, anxiety, depression, decision satisfaction).
- Cost-effectiveness modelling.

Statistical treatment of data for this paper was restricted to description of numbers and percentages responding who were eligible, recruited and screened, with outcomes of the screening.

### Cost-effectiveness modelling based on UKLS pilot data

A full UKLS trial was planned to follow the pilot: this would have randomised an additional 28 000 subjects but was not funded. The pilot UKLS trial was not powered to evaluate mortality reduction and this short follow-up period precluded adopting the conventional approach to trial evaluation, namely, the measurement of long-term costs and outcomes in the test and control arms, and the comparison thereof. Thus, the observational element of the economic evaluation was restricted to those events and findings that occurred within the active trial period. The detailed modelling methodology for calculating the UKLS cost effectiveness is described in the online supplementary section.

## Results

### Recruitment

From the 247 354 people sent questionnaires, 148 608 (60.1%) were non-responders (no questionnaire returned), 22 788 (9.2%) were negative responders (non-participation questionnaire returned) and 75 958 (30.7%) were positive responders (questionnaire returned; willing to participate). Of the positive responders, 8729 (11.5%) were classified by the LLP_v2_ as high risk, with a risk of ≥5% of developing lung cancer over the next 5 years (mean LLP_v2_ risk score=8.8% versus 1.0% for the low risk group). A total of 5967/8729 (68.4%) high-risk responders returned the second questionnaire and agreed to participate; 1291 of these were subsequently excluded for the following reasons: they did not meet the inclusion criteria; they had not completed the eligibility questionnaire correctly; they replied after maximum trial recruitment numbers had been reached; or they were unable to give fully informed consent. Five hundred and eighty-two individuals either changed their mind or failed to attend clinic, and a further 33 attended the recruitment clinic but declined to consent. No stratification criteria were used. In total, 4061 individuals (5.3% of all positive responders, and 46.5% of all high-risk positive responders) consented and were recruited into the UKLS.

**Figure 3:**
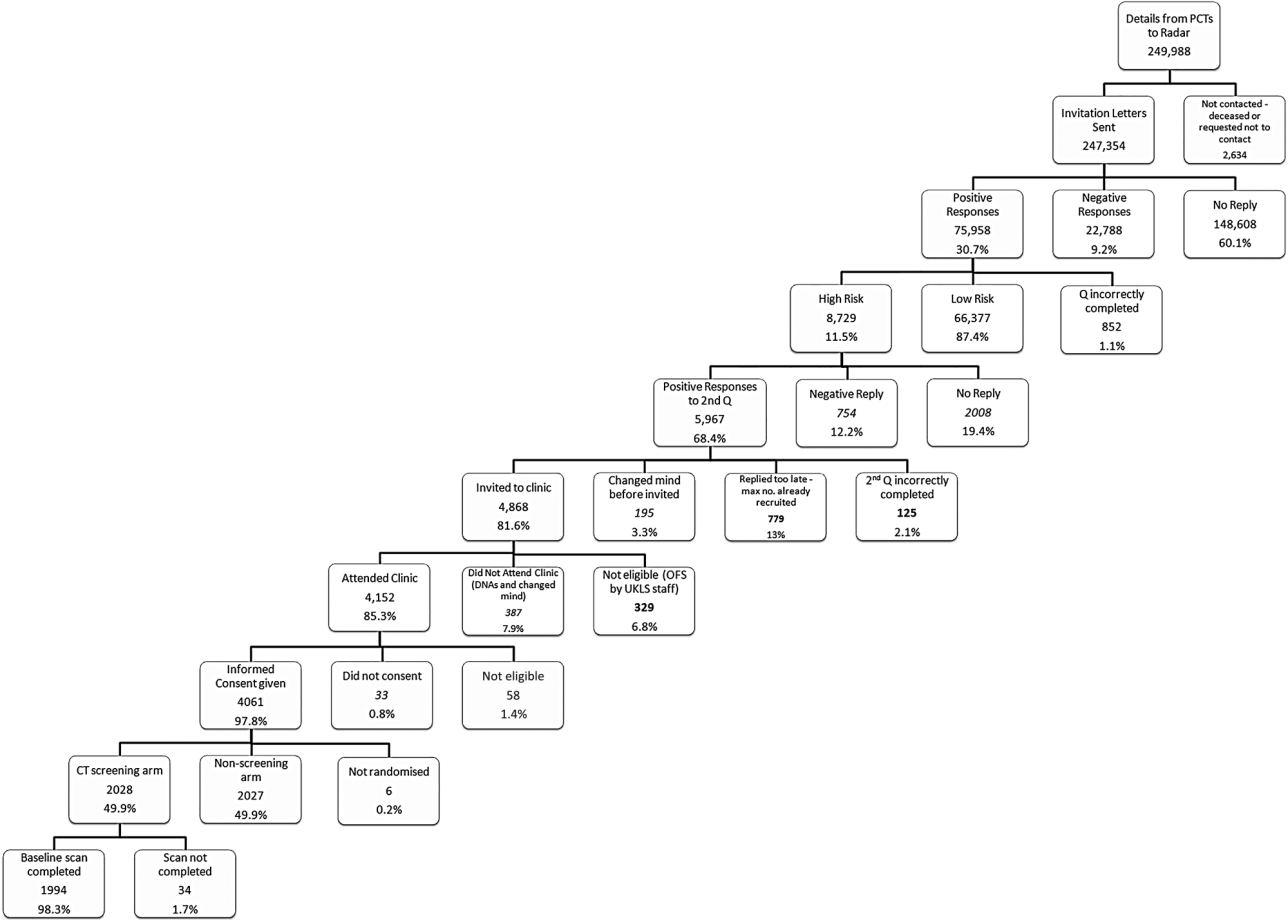
Participant flow from initial contact to CT screening.

**Figure 4:**
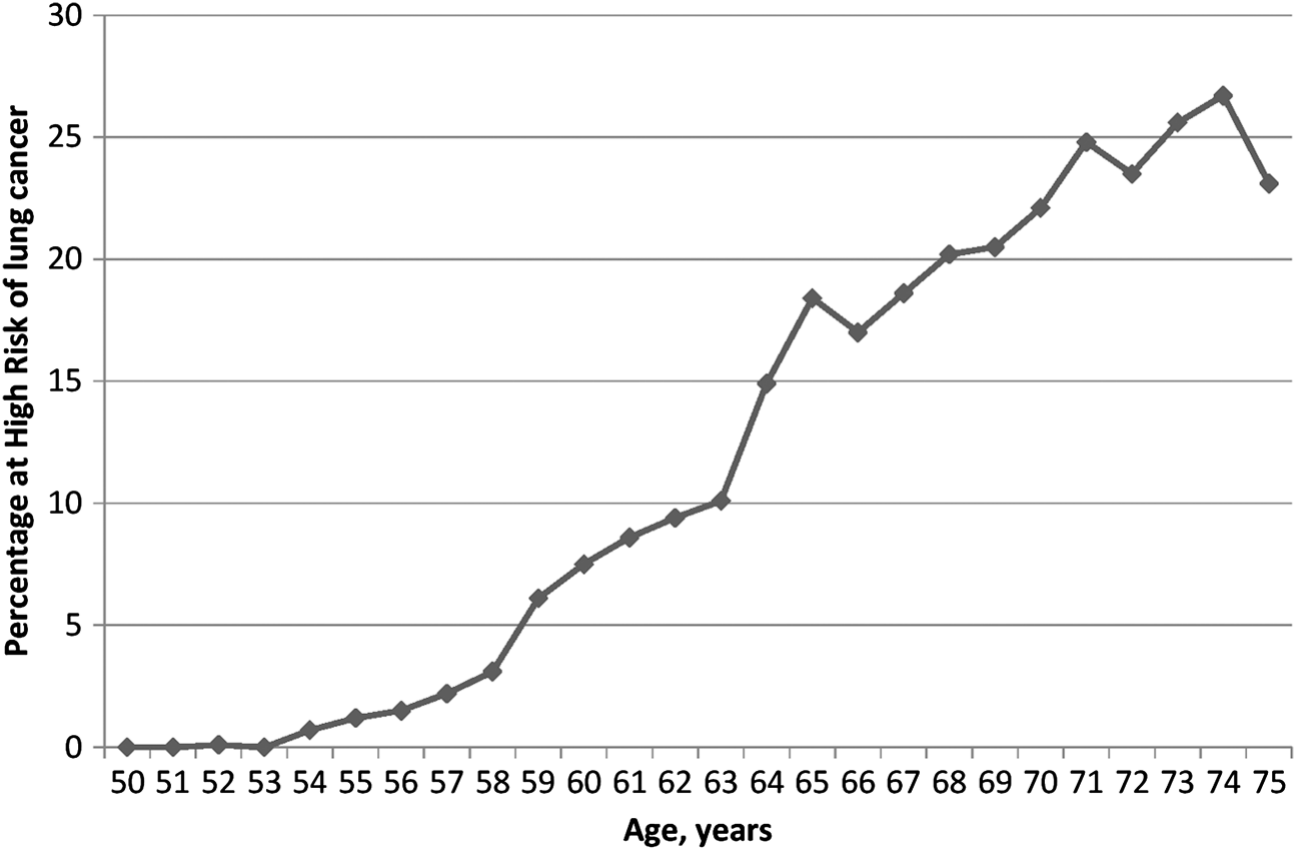
Percentage of UK Lung Cancer Screening (UKLS) positive responders (n=75 958) with a Liverpool Lung Project (LLP_v2_) risk of >5%, by individual age.

Figure 3 shows the participant flow from invitation through to randomisation. Figure 4 provides the percentage of UKLS positive responders (n=75 958) with an LLP risk of ≥5%, by individual age; there was a steady increase with age in the percentage of people at high risk, with very low numbers at age 50–54.

A total of 73 934 (97.3%) of the positive responders gave information about their smoking habits: 43.4% were never smokers, 14.7% current smokers and 39.3% ex-smokers. A total of 22 024 (96.6%) of the 22 788 negative responders gave information on smoking habit: 51.3% were never smokers, 9.1% current smokers and 36.2% ex-smokers.

### CT screened participants

A total of 1994 participants underwent CT by July 2014; 42 participants (2.1%) were diagnosed with lung cancer, 34 (1.7%) at baseline and 8 (0.4%) were diagnosed within 12 months with follow-up CT. Characteristics of individuals in both arms of the trial were very similar (table 1). The date of censoring was 30 September 2014.

**Table 1:** Demographic, risk and medical characteristics of n=4055 individuals randomised to the UK Lung Cancer Screening (UKLS) intervention (CT screen) and control (non-screen) trial arms

	Total n=4055	
	Screen arm (n=2028)	Control arm (n=2027)
Male:female ratio	1529:499 (3.06:1)	1507:520 (2.90:1)
North:south ratio	1023:1005 (1.02:1)	1023:1004 (1.02:1)
Mean (SD) age, years	67.1 (4.1)	66.9 (4.1)
Median age, years	67	67
Median IMD rank*	17 374	17 704
Mean (SD) LLP_v2_ score	8.87 (5.12)	8.83 (4.71)
Median LLP_v2_ score	7.11	7.35
Never smokers	2 (0.1)	0 (0%)
Current smokers	777 (38.3%)	791 (39.0%)
Ex-smokers	1249 (61.6%)	1236 (61.0%)
Smoking duration 10–19 years†	117 (5.8%)	116 (5.7%)
Smoking duration 20+ years†	1895 (93.4%)	1907 (94.1%)
Smoking duration unknown†	14 (0.7%)	4 (0.2%)
% Asbestos exposed	763 (37.6%)	763 (37.6%)
% with history of respiratory disease‡	1056 (52.1%)	1023 (50.5%)
% with history of blood cancer§	26 (1.28%)	31 (1.53%)
% with history of solid tumour¶	378 (18.6%)	396 (19.5%)
Total % with family history of lung cancer	498 (24.6%)	554 (27.3%)
% with family history of lung cancer <60 years	215 (10.6%)	215 (10.6%)
% with family history of lung cancer >60 years	283 (14.0%)	339 (16.7%)
Family history of other cancer (not lung)**	1026 (50.6%)	1019 (50.3%)

*Index of Multiple Deprivation (IMD) rank (https://www.gov.uk/government/collections/english-indices-of-deprivation).

†All smoking duration figures refer to current and ex-smokers combined.

‡Asthma, bronchitis, TB, pneumonia, COPD or emphysema.

§Leukaemia or lymphoma, including Hodgkin's.

¶Cancers of brain, head and neck, oesophagus, breast, colon or ‘other’.

**Cancers of brain, head and neck, oesophagus, breast, colon or ‘other’.

LLP, Liverpool Lung Project.

### Nodules and management

There were 1015 (50.9%) subjects with category 2–4 nodules; 479/1994 (24%) subjects with category 2 nodules underwent a 12-month repeat scan. Seven of 479 (1.5%) were referred to the MDT, of whom 1/479 (0.2%) was diagnosed with lung cancer (table 2).

**Table 2:** Numbers of UKLS individuals in each Nodule category; MDT referral and the number of confirmed lung cancers.

Nodule category (management)	Cat 1 (discharged)	Cat 2 (repeat scan at 12 months)	Cat 3 (repeat scan at 3 months then 12 months)	Cat 4 (immediate MDT referral)	Total
Number in category	979	479	472	64	1994
Number referred to MDT	0 (N/A)	7	43	64	114
Number of confirmed lung cancers	0 (N/A)	1	9	32	42

MDT, multidisciplinary team.

Four hundred and seventy-two subjects (23.7%) had category 3 nodules and underwent a 3-month interval CT. Forty-three of the participants (9.1%) were referred to the MDT. A total of nine (1.9%) lung cancers were diagnosed; two at month three and seven at the 12-month repeat scan.

Of 64 participants with category 4 nodules who were referred directly to the MDT, 32 (50%) had lung cancer (table 3).

**Table 3:** Lung cancer diagnosed in pilot UK Lung Cancer Screening (UKLS)

UKLS case No	Baseline nodule category*	Sex	Age	TNM	Final Stage	Diagnosis	Treatment
1	4	M	59	pT1a pN0	IA	Adenocarcinoma	Surgery
2	4	M	66	pT1a pN0	IA	Adenocarcinoma	Surgery
3	4	M	66	pT1a pN0	IA	Adenocarcinoma	Surgery
4	4	M	55	pT1b pN0	IA	Adenocarcinoma	Surgery
5	4	M	63	pT1a pN0	IA	Adenocarcinoma	Surgery
6	4	F	64	pT1a pN0	IA	Adenocarcinoma	Surgery
7	4	M	67	pT1b pN0	IA	Small cell carcinoma	Surgery/chemotherapy
8	4	M	62	pT1a pN0	IA	Squamous cell carcinoma	Surgery
9	4	M	68	pT1b pN0	IA	Squamous cell carcinoma	Surgery
10	4	M	67	pT1a pN0	IA	Squamous cell carcinoma	Surgery
11	4	M	73	pT1b pN0	IA	Squamous cell carcinoma	Surgery
12	4	M	71	pT1a pN0	IA	Adenocarcinoma	Surgery
13	4	M	72	cT1b cN0 cM0	IA	Adenocarcinoma	Radiotherapy
14	4	M	64	pT1b pN0	IA	Adenocarcinoma	Surgery
15	4	M	68	pT1a pN0	IA	Adenocarcinoma	Surgery
16	4	M	74	cT1a cN0 cM0	IA	Bronchogenic carcinoma	Palliative
17	4	M	69	pT1a pN0	IA	Squamous cell carcinoma	Surgery
18	4	M	70	pT2a pN0	IB	Adenocarcinoma	Surgery
19	4	M	67	pT2a pN0	IB	Adenocarcinoma	Surgery
20	4	M	68	pT2a pN1	IIA	Squamous cell carcinoma	Surgery/chemotherapy
21	4	F	67	pT1a pN1	IIA	Squamous cell carcinoma	Surgery/chemotherapy
22	4	F	64	pT2b pN0	IIA	Adenocarcinoma	Surgery/chemotherapy
23	4	F	73	pT2a pN1	IIA	Small cell carcinoma	Surgery/chemotherapy
24	4	M	63	pT1a pN1	IIA	Adenocarcinoma	Surgery/chemotherapy
25	4	M	75	pT2a pN1	IIA	Squamous cell carcinoma	Surgery/chemotherapy
26	4	M	64	pT2b pN0	IIA	Carcinoid	Surgery
27	4	M	68	pT1a pN2	IIIA	Adenocarcinoma	Surgery
28	4	F	69	pT1b pN2	IIIA	Adenocarcinoma	Surgery/chemotherapy
29	4	M	63	cT1a cN2 cM0	IIIA	Small cell carcinoma	Chemotherapy
30	4	F	60	pT3 pN0	IIB	Squamous cell carcinoma	Surgery/radiotherapy
31	4	M	66	cT4 cN3 cM1b	IV	Adenocarcinoma	Chemotherapy/radiotherapy
32	4	M	64	cT3 cN2 cM1b	IV	Squamous cell carcinoma	Palliative
33	3 (3 months)	M	68	pT1a pN0	IA	Adenocarcinoma (two primaries)	Surgery
34	3 (12 months)	M	69	pT1a pN0	IA	Adenocarcinoma	Surgery
35	3 (12 months)	M	61	pT1a pN0	IA	Squamous cell carcinoma	Surgery
36	3 (12 months)	M	70	pT1a pN0	IA	Adenocarcinoma	Surgery
37	3 (3 months)	F	70	pT1aNx†	IA	Adenocarcinoma	Surgery
38	3 (12 months)	M	66	pT1b pN0	IA	Squamous cell carcinoma	Surgery
39	3 (12 months)	F	69	cT1a cN0 cM0	IA	Adenocarcinoma	Radiotherapy
40	3 (12 months)	M	71	pT1a pN0	IA	Adenocarcinoma	Surgery
41	3 (12 months)	F	75	cT4 cN2 cM1b	IV	Adenocarcinoma	Surgery (non-pulmonary) Radiotherapy/chemotherapy
42	2	F	66	pT1a pN0	IA	Adenocarcinoma	Surgery

*Baseline nodule category—category 4 referred to MDT at baseline, category 3 referred for repeat CT at 3 months and 12 months, category 2 referred for 12 month repeat CT.

†Participant underwent wedge resection. Clinical stage was cT1a cN0 cM0.

MDT, multidisciplinary team.

### Diagnostic workup and false positives

In the UKLS, we defined false positives as those requiring further diagnostic investigation more immediately than a repeat annual screen, but who subsequently did not have lung cancer. This is because future screening programmes are likely to include annual or biennial screens. Overall, 951/1994 (47.7%) of subjects underwent at least one further CT after the initial screen.

For complete clarity, the proportion of false positive tests is now provided in two ways, which allows an appreciation, in a patient-centred approach, of the variable impact on the subject in a trial or the patient in a programme. A ‘false positive’ that mandates referral to the lung cancer MDT will usually be associated with significant psychological distress and additional more or less invasive investigations with, in some cases, definitive treatment. An individual with a false positive test so defined is thus more likely to suffer harm than one defined in a different way; that is, those subjects who are recalled solely for further CT imaging to clarify the nature of a nodule. The latter is best termed ‘interval imaging rate’ and may, in screening programmes, merely mean continuing in the programme rather than referral to the MDT. For this reason, all category 3 lesions without cancer are reported separately as false positives warranting interval imaging. Category 2 findings are not classified as false positives warranting recall as the cancer rate was found to be so low in this study that interval imaging would not be recommended.

Thus on examining the number of UKLS participants referred to the MDT clinic, the false-positive rate is 3.6% (114-42/1994=3.6); whilst the interval imaging rate is 23.2% (472-9/1994).

In total, 114/1994 (5.7%) participants were referred to the MDT, of whom 42 (2.1% of all screened) had lung cancer.

### Pathology

Of the 42 screen-detected cancers, there were 25 adenocarcinomas, 12 squamous cell carcinomas, 3 small cell carcinomas, 1 typical carcinoid, and 1 bronchogenic carcinoma. Twenty-eight of 42 (66.7%) lung cancers were detected at stage I and 8/42 (19%) at stage II (table 3). In total, 36/42 (85.7%) were stage I or II.

### Treatment

Thirty-five of 42 subjects (83%) had surgery as their primary treatment, with eight having adjuvant chemotherapy. Thirty-three of the 36 patients with stage I and II lung cancer had surgery (91.6%) and a further two had radical radiotherapy (total 97%). In the seven patients who did not undergo resection, lung cancer diagnoses were made radiologically in one individual and via tissue biopsy in six patients. Four patients with benign disease had surgery (benign resection rate of 10.3% (4/39)). Details of the UKLS patients with lung cancer with final pathology, TNM, stage and management are shown in table 3.

### Cost effectiveness

A detailed description of the results of the health economics modelling is given in the online supplementary section. The baseline estimate for the incremental cost-effectiveness ratio (ICER) of once-only CT screening relative to symptomatic presentation, under the UKLS protocol, was £8466 per quality adjusted life year (QALY) (CI £5542 to £12 569).

## Discussion

The UKLS pilot has demonstrated that, by using a population-based approach and a validated risk assessment model, it is possible to detect lung cancer at an early stage using LDCT screening. Over 85% of lung cancers detected were stage I or II, and over 90% of these cases were able to have potentially curative treatment.

Overall, there was a 1.7% prevalence of lung cancer at baseline which is higher than that reported by the NLST^1^ or NELSON^11^ ^12^ trials. This reflects the relatively high minimum risk threshold using the LLP_v2_ risk model (≥5% over 5 years).

The postal questionnaire approach achieved a 30.7% positive response rate to the initial mailing, a good response rate for a clinical trial; however, only 3.5% of the total met the eligibility criteria. Better targeting of individuals with a high risk will be needed for a screening programme.^13^

UKLS has demonstrated that a volumetry-based nodule management algorithm accurately selected participants for referral to the MDT, resulting in one of the lowest reported rates of surgical procedures for benign disease (10.3%). The trial has also confirmed the very low rate of malignancy in small nodules shown in NELSON.^14^ Only one (0.2%) participant with nodules between 15 and 50 mm^3^ (or 3–5 mm diameter, if volumetry not possible) was proved to have lung cancer within a 12-month period. The rate of malignancy was thus well below the baseline risk in screening populations.

We have defined the terms false positive and interval imaging rates, thus encapsulating the concept of the level of harm to the participants. This distinction informs the clinician of the rates potentially associated with significant harm and the rate associated with the need for follow-up imaging, which may be part of future screening programmes. The UKLS false-positive rate was 3.6% and the interval imaging rate was 23.2%.

Our interval imaging rate corresponds to the false-positive rate reported in NLST (23.3%), in which a positive finding on CT was any non-calcified nodule at least 4 mm in diameter.^1^ In the NELSON trial lung nodules with a volume >500 mm^3^ or those with a volume-doubling time <400 days were regarded as positive tests. Horeweg *et al*^11^ ^15^ reported that over three screening rounds, 458 (6%) of the 7582 participants screened had a positive result and 200 (2.6%) were diagnosed with lung cancer; 3.6% of all NELSON participants (273 out of 7582) had a false-positive screening result. This corresponds to our definition of false-positive rate. However, at the first round in NELSON, 19% of cases had indeterminate findings, which required a repeat scan to assess growth.^11^

The modelled ICER of once-only CT screening under the UKLS protocol was of the order of £9000 per QALY. This is broadly consistent with the ICERs of other recent studies, once allowance is made for differential efficiency of screening protocols. The ICER would be less favourable if there were substantial overdiagnosis but better if smoking cessation were improved. The prevalence of lung cancer was consistent with the risk status of the UKLS recruits, so substantial overdiagnosis is unlikely.

The comparison of the NLST and UKLS cost-effectiveness approaches is outlined in table 4. The UKLS ICER is about one-fifth of the NLST's costs ($81 000), mainly explained by the differences in unit costs and in the intensity of resource use to detect and manage the same proportion of cancers. The UKLS was a single screen compared with the NLST's three annual screens but both gave similar yields of lung cancer.

**Table 4:** Comparison of cost-effectiveness approaches used in the National Lung Cancer Screening Trial (NLST) and UK Lung Cancer Screening(UKLS)

	UKLS	NLST^16^	Consequence (USA compared with the UK)
1	Yield=2.1% of persons screened	Yield=2.0% of persons screened	Similar yield
2	Single prevalence screen. Screening and workup costs per person=£212=$327 at current exchange rates (7 July 2015)	3 screens to produce similar yield Screening and workup costs per person screened=$1965 (table 2)	Far more resources devoted to initial detection in USA Any US resource has a higher unit cost
3	Net treatment costs per person (screen-detected vs no screening)=£60=$92	Net treatment costs per person (screen detected vs no screening)=$175	US costs treatment costs higher
4	Costs of patient time and travel to appointments are NOT included in total costs: the evaluation adopts an NHS perspective, as recommended by NICE	Costs of patient time and travel to appointments are included in total cost: the evaluation adopts a social perspective, as recommended by the US Panel on Cost-Effectiveness in Health and Medicine	Inclusion of patient costs makes US screening and management appear to be more expensive (and less cost effective)
5	Outcome estimate calculations based on life table survival estimates	Outcome estimates calculations based on life table survival estimates	Similar life table survival estimation method used in both trials
6	Incremental quality adjusted life years (QALYs) gained per person screened=0.03	Incremental QALYs gained per person screened=0.02 overall, but 0.03 in the age range 60–69	Gains per person screened appear essentially similar
7	UKLS modelling based on 1 year £8466 per QALY gained (CI £5542 to £12 569) $13 071 per QALY gained (CI $8556 to $19 405)	NLST $81 000 per QALY gained (5% CI 52 000 to 186 000). Calculations based on quintiles 4 and 5, accounts for a significantly higher proportion of the lung cancer deaths: costings: 4th quintile $32,000/QALY; 5th quintile $52,000/QALY (ie, £20,921; £33,996)	Allowing for the fact that the medical care in the USA is more expensive than the UK, the NLST ICER would be at least halved, if the screening had been confined to the two highest risk quintiles

NICE, National Institute for Health and Care Excellence.

One of the limitations of our analysis is that the mortality benefit had to be estimated from NLST. Interestingly, estimates from both NLST and UKLS were similar, despite the fact that UKLS targeted a higher risk group (table 4).

The main shortcoming of the UKLS is that it is a pilot study and is not powered for a long-term mortality comparison in isolation. However, it is planned to pool the UKLS data, including mortality data, from both arms with the European screening trials,^17^ including NELSON, in 2016.

In summary, the UKLS pilot has shown that CT screening in the UK is possible using a risk prediction model that avoids the selection of people at very low risk who are unlikely to benefit,^18^ and a nodule management algorithm that effectively manages indeterminate CT findings, yet detects a high number of early stage lung cancers.

Taking the UKLS pilot trial data in consort with the NLST mortality data, health economics modelling showed a promising ICER. However, if CT screening is adopted, efforts to maximise cost effectiveness should be made, such as integrated smoking cessation. The results from the NELSON trial and the pooled UKLS and NELSON trial data will likely influence the decision to undertake lung cancer screening in the UK.^19^ ^20^

## Supplementary Material

Web UKLS_COST_EFFECTIVENESS

Web UKLS_NODULE_MANAGEMENT
