# Supplementary material for: UK Lung Cancer RCT Pilot Screening Trial: baseline findings from the screening arm provide evidence for the potential implementation of lung cancer screening
Source: Thorax. 2015 Dec 8;71(2):161–70. doi: 10.1136/thoraxjnl-2015-207140 (PMC4752629; doi:10.1136/thoraxjnl-2015-207140)
Supplement: Web UKLS_COST_EFFECTIVENESS [file thoraxjnl-2015-207140-s1.pdf]

Supplementary Section

Cost effectiveness – modelled for UKLS

Originally a full UKLS trial was planned to follow the pilot. This would have randomised an additional 28,000 subjects but was not funded. The pilot UKLS trial was not powered to evaluate mortality reduction and the short follow-up period precluded adopting the conventional approach to trial evaluation, namely, the measurement of long-term costs and outcomes in both the test and the control arms, and the comparison thereof. Of necessity, the observational element of the economic evaluation was restricted to those events and findings that occurred within the active trial period. Observable costs which accrued in the active period were those of (i) screening the population, (ii) re-screening or investigating patients with suspicious findings according to the trial protocol,[1] (iii) diagnostic work-up and treatment for the detected cancers. An allowance was made for the costs avoided by not having to treat cancer that otherwise would have presented in the future, had they not been screen-detected. All costs were expressed in UK pounds sterling at 2011-12 prices. For most events, the unit costs of procedures were 2011-12 National Health Service tariffs or reference costs, as classified according to the appropriate Healthcare Resource Groups (HRG) coding.[2]

Given the number of events recorded during UKLS, and the unit costs of each event, the mean gross current cost of the trial amounted to £687,617. This total comprised (i) £282,490 for the CT scans (ii) £72,592 for work-up via the MDTs and (iii) £332,534 for the treatment of the detected cancers. As trial invitation and selection had been configured for recruitment and research purposes, we modelled an invitation protocol appropriate for a screening programme. This added 13 per cent to the total programme cost, yielding a gross total cost of £754,877. Against this total, we offset the estimated future treatment costs avoided as a result of screening, namely, the cost of managing the detected cancers, which would otherwise have presented symptomatically. This offset resulted in a net programme cost of £565,498, or £13,464 per lung cancer detected. To construct confidence intervals we assumed normality in all unit costs and converted the inter-quartile ranges of unit costs for the principal event types into standard deviations. We then re-estimated the net cost

calculation by simulation, using distributions governed by standard deviations about the mean unit costs (Palisade @RISK with 20,000 iterations). The simulation produced a 95 per cent confidence interval for net programme costs of £362,564 to £769,309.

The brief duration of UKLS precluded the measurement of life year gains from screening. Therefore, the benefits of screening were estimated by simulation, comparing the expected survival of each of the screen-detected cases, given age, sex and stage at detection, and expected survival following symptomatic presentation, as would have been the case in the absence of screening. The simulation employed published survival data from other studies and an existing survival model based on life tables. [3] From the characteristics of the cancer patients identified by UKLS, we estimated an average gain as a result of early detection and treatment of 3.3 (CI 2.6 to 3.9) life years per cancer, undiscounted, or 2.1 (1.7 to 2·5) life years, discounted. Again, trial brevity did not permit long-term assessment of quality of life. However, as most of the gains from a screening programme accrue to those treated successfully for early stage cancers, Health Related Quality of Life (HRQL) pre- and post-screening must be essentially similar to population norms. For the UK, HRQL norms for the sexes/ages at which simulated deaths from cancer would have occurred lie in the range 0·71 to 0·78 relative to perfect health.[4] Adjusting life year gains for each cancer detected by the HRQL coefficient for the patient's expected age at death transformed the predicted total gain of 89.4 discounted life years into 66.8 discounted Quality Adjusted Life Years (QALYs), or 0·03 QALYs per person screened. In NLST, the rate of lung cancer mortality was 0·0024 per year in the CT arm and 0·0030 per year in the control arm. Over ten years this would amount to 0·03 years of life saved per person invited. The greater lung cancer risk in the UKLS group and conversion to a denominator of persons screened rather than invited, would increase this value whereas quality adjustment and discounting would decrease it, thus arriving at roughly the same figure. Thus these estimates are consistent with the results of the major randomised trial evidence.

The baseline estimate for the Incremental Cost Effectiveness Ratio (ICER) of once-only CT screening relative to symptomatic presentation, under the UKLS protocol

and with conservative assumptions, was £8,466 per QALY gained (CI £5,542 to £12,569).

1. Baldwin DR, Duffy SW, Wald NJ, Page R, Hansell DM, Field JK: **UK Lung Screen (UKLS) nodule management protocol: modelling of a single screen randomised controlled trial of low-dose CT screening for lung cancer.** *Thorax* 2011, **66:**308-313.
2. **Unit Costs of Health and Social Care 2011. Personal and Social Services Research Unit (PSSRU); 2011.** In *(CDC), Centers for Disease Control and Prevention* (Curtis L ed. Personal Social Services Research Unit, Cornwallis Building, The University of Kent, Canterbury, Kent CT2 7NF, UK.; 2011.
3. Whynes DK: **Could CT screening for lung cancer ever be cost effective in the United Kingdom ?** *Cost Eff Resour Alloc* 2008, **6:**5.
4. Kind P, Hardman G, Macran S: *UK population norms for EQ-5D (Discussion Paper 172).* York: Centre for Health Economics, University of York; 1999.
