# Supplementary material for: UK Lung Cancer RCT Pilot Screening Trial: baseline findings from the screening arm provide evidence for the potential implementation of lung cancer screening
Source: Thorax. 2015 Dec 8;71(2):161–70. doi: 10.1136/thoraxjnl-2015-207140 (PMC4752629; doi:10.1136/thoraxjnl-2015-207140)
Supplement: Web UKLS_NODULE_MANAGEMENT [file thoraxjnl-2015-207140-s2.pdf]

UKLS NODULE MANAGEMENT PROTOCOL

Category 1: Benign nodules fulfilling one of the following criteria: a benign pattern of calcification, presence of fat, nodules measuring <3mm in diameter or volume <15mm^3. Or: Intrapulmonary lymph nodes fulfilling the following criteria: they lie within 5 mm of the pleura, are <8mm in diameter, are smooth bordered and ovoid and have at least one interlobular septum or linear opacity radiating from their surface.

Category 2: If solid and intraparenchymal, volume of 15-49mm^3 or maximum diameter of 3-4·9mm, if nodules could not be segmented by volumetry software. If solid and pleural or juxtapleural, a maximum diameter of 3-4·9mm. If non-solid or part solid, a maximum diameter of the ground glass component of 3-4·9 mm. If part-solid, the solid component has a diameter of <3mm and/or volume of <15mm^3.

Category 3**:** If solid and intraparenchymal, a volume of 50-500mm^3 or diameter of 5-9·9mm if nodules could not be segmented by volumetry software. If solid and pleural or juxtapleural, a diameter 5-9·9mm. If non-solid or part-solid, a diameter of the ground-glass component of >5mm. If part solid, the solid component has a volume of 15-500 mm^3 or has a maximum diameter of 5–9·9 mm.

Category 4**:** If solid and intraparenchymal, a volume >500mm3 or diameter of ≥10mm if nodules could not be segmented by volumetry software. If solid and pleural or juxtapleural, a diameter of ≥10 mm. If part solid, the solid component has a diameter of ≥10mm or has a volume >500mm^3

Nodules were managed as follows:

No nodules or Category 1 nodules: No further action required.

Category 2 nodules: Follow up CT scan at 12 months.

Category 3 nodules: Follow up CT scan at 3 months and (if required) subsequently 12 months from baseline.

Category 4 nodules: Referral to Multidisciplinary Team (MDT).

Where follow up scans (at 3 or 12 months) were performed, the volume doubling time (VDT) of the nodule was calculated. VDTs were designated as: < 400 days or ≥400 days.
